# Conventional and Microfluidic Methods for the Detection of Nucleic Acid of SARS-CoV-2

**DOI:** 10.3390/mi13040636

**Published:** 2022-04-17

**Authors:** Weidu Song, Taiyi Zhang, Huichao Lin, Yujing Yang, Gaozhen Zhao, Xiaowen Huang

**Affiliations:** State Key Laboratory of Biobased Material and Green Papermaking, Department of Bioengineering, Qilu University of Technology (Shandong Academy of Sciences), Jinan 250300, China; 10431200892@stu.qlu.edu.cn (W.S.); 10431201118@stu.qlu.edu.cn (T.Z.); 1043119736@stu.qlu.edu.cn (H.L.); 10431211246@stu.qlu.edu.cn (Y.Y.); 10431210986@stu.qlu.edu.cn (G.Z.)

**Keywords:** SARS-CoV-2, nucleic acid testing, polymerase chain reaction, isothermal amplification, microfluidic, CRISPR–Cas systems

## Abstract

Nucleic acid testing (NAT) played a crucial role in containing the spread of SARS-CoV-2 during the epidemic. The gold standard technique, the quantitative real-time polymerase chain reaction (qRT-PCR) technique, is currently used by the government and medical boards to detect SARS-CoV-2. Due to the limitations of this technology, it is not capable of meeting the needs of large-scale rapid detection. To solve this problem, many new techniques for detecting nucleic acids of SARS-CoV-2 have been reported. Therefore, a review that systematically and comprehensively introduces and compares various detection technologies is needed. In this paper, we not only review the traditional NAT but also provide an overview of microfluidic-based NAT technologies and summarize and discuss the characteristics and development prospects of these techniques.

## 1. Introduction

In late 2019, a new coronavirus, posing a significant threat to human life, was discovered. The International Committee on Taxonomy of Viruses (ICTV) officially named the virus the severe acute respiratory syndrome coronavirus 2 (SARS-CoV-2). In the early stages of the outbreak, China first identified the virus [1] and found that it could be transmitted in the population [2]. It is reported that the main clinical features of COVID-19 are fever, fatigue, cough, and loss of smell [3]. Severe cases might rapidly progress to acute respiratory distress syndrome (ARDS), septic shock, metabolic acidosis bleeding, and coagulation dysfunction [4,5].

Early diagnosis is one of the basic measures to contain the spread of SARS-CoV-2 [6]. When SARS-CoV-2 infects humans, it multiplies in parts of the body such as the nasal cavity, pharynx, and lower respiratory tract [7]. Therefore, after collecting human nasopharyngeal swabs, sputum, and other samples for nucleic acid testing (NAT), we are capable of determining whether the human body is infected with SARS-CoV-2 [8,9].

**Figure 1:**
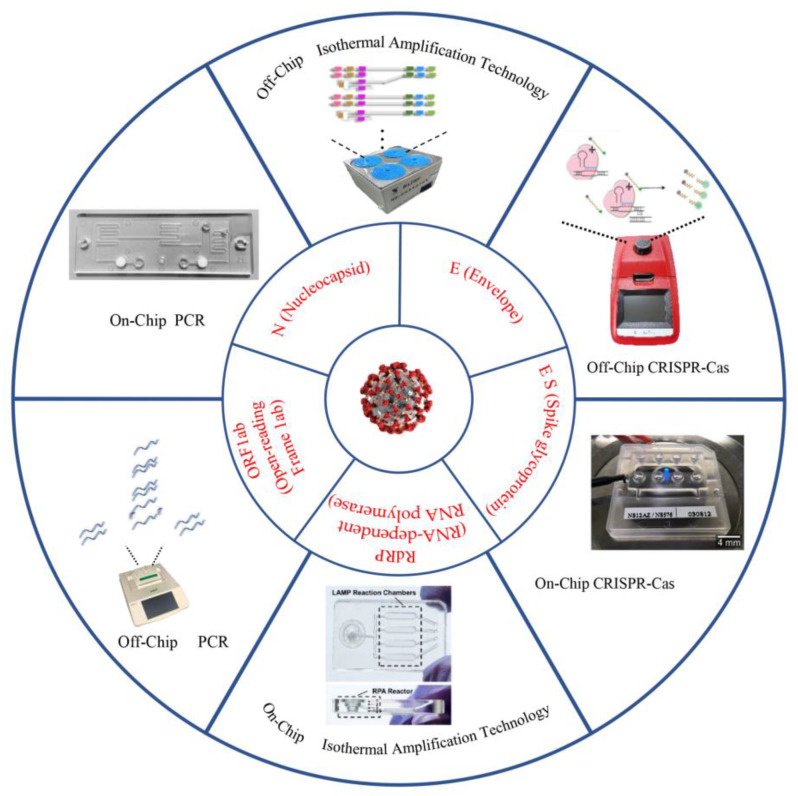
Some essential nucleic acid detection techniques with the potential for detecting SARS-CoV-2 [16,17,18,19]. (Copyright © 2021 Elsevier B.V. All rights reserved.) (Copyright © 2020 The Healthcare Infection Society. Published by Elsevier Ltd. All rights reserved.) (Copyright © 2021 Elsevier B.V. All rights reserved).

Mass NAT of the population is essential for early diagnosis [10]. It has become an indispensable tool for epidemic prevention and containment in many countries. However, the existing NAT is mainly conducted by using polymerase chain reaction (PCR) technology with some problems in practical application. First of all, nucleic acid detection requires a long process. Extracting samples from patients, storing and delivering them to the testing center, and applying for offline result reports takes up a great amount of time. Second, the productivity of current biotechnology products (enzymes, primers, and buffers) is not sufficient to meet the requirements for extensive testing of the population. Third, companies produce an insufficient amount of test kits commercially [11]. Microfluidic technology may be able to solve the above problems. In recent years, researchers have developed miniature devices for detecting pathogens. They create new solutions to healthcare challenges [12,13]. The microfluidic detection chip is capable of easily extracting samples, performing detection, and completing laboratory operations. Microfluidics allows high-throughput testing of samples with fewer biological reagents, and the use of microfluidics for NAT will solve the difficulty of shortage of biological reagents [14,15]. In this review, we discuss the feasibility and latest progress of microfluidic detection equipment from several aspects. In addition, we also compared and analyzed the advantages and disadvantages of microfluidic detection chips and bench tests and looked forward to the prospects of microfluidic technology in the area of testing (Figure 1).

## 2. Off-Chip Nucleic Acid Detection Method for SARS-CoV-2 Detection

The researchers sequenced the genome of the original SARS-CoV-2 virus after the outbreak [20]. They screened out unique nucleic acid sequences that provided a theoretical basis for nucleic acid detection. According to reports, testers conduct NATs on samples, usually through nucleic acid amplification tests (NAATs), to detect unique viral RNA sequences in Nucleocapsid (N), Spike glycoprotein (S), Envelope (E), and RNA-dependent RNA polymerase (RdRp) [21]. There are many methods for NAT: quantitative real-time polymerase chain reaction (qRT-PCR), isothermal amplification reaction [22], and CRISPR–Cas technology. Overall, either method of detecting SARS-CoV-2 should pursue the detection speed and limit of detection (LOD) based on the guarantee of accuracy.

### 2.1. Nucleic Acid Detection Based on PCR Technology

Although PCR is the gold standard [23], it is challenging to meet the urgent need for detection in the reality of irregularly breaking out and constantly mutating viruses [24]. Some researchers have proposed a simple, cheap, and fast alternative. In this method, the oropharyngeal swab was heated at 98 °C for 5 min and then cooled at 4 °C for 2 min before performing the qRT-PCR reaction [25]. The positive detection rate of SARS-CoV-2 reached 97.4% without false negatives. However, this method may degrade part of the RNA during heating. Therefore, we use this method only without nucleic acid purification reagents. In fact, in the face of complex and diverse samples, the sensitivity, accuracy, and repeatability of many real-time polymerase chain reaction (RT-PCR)-based nucleic acid detection kits are not ideal, which prevents them from being used in large-scale diagnosis. To make it suitable for large-scale clinical diagnosis, Tao et al. compared the detection results of droplet digital PCR (ddPCR) and PCR in terms of sensitivity, specificity, and accuracy and found that the detection performance of ddPCR was better than PCR [23]. Nevertheless, because of its high detection cost, it is not suitable for wide applications.

In an environment with a severe shortage of cotton swabs and personal protective equipment, saliva is selected as a test sample by testing personnel. Although the sensitivity of saliva as a diagnostic specimen is less than that of nasopharyngeal swabs (NPS), it reduces exposure to risk, consumables, and discomfort [26]. La Rosa et al. detected SARS-CoV-2 RNA in wastewater samples using RT-PCR [27], but it was not clear whether the SARS-CoV-2 RNA was infectious. Bivins et al. used virus culture technology to cultivate SARS-CoV-2 in wastewater while using qRT-PCR technology to detect SARS-CoV-2 RNA in wastewater. The study measured the infectivity of SARS-CoV-2 and the persistence of RNA signals in water and wastewater. The experimental results showed that SARS-CoV-2 RNA was more durable than infectious SARS-CoV-2, which indicated that environmental testing of RNA alone could not confirm the risk of infection [28]. The detection of SARS-CoV-2 in the air in hospital wards is of great significance for improving the management of healthcare facilities and implementing public safety measures compared to other regions. Other experimental studies have shown that SARS-CoV-2 RNA exists in the air of hospitals with patients. To reduce the risk of infection, medical personnel working in these areas are required to implement strict personal protection and systematic disinfection [29].

In conclusion, researchers can detect viruses in resource-constrained testing environments by extending DNA denaturation time and using saliva as a sample (Table 1). Nucleic acid testing in densely populated environments can remind people to implement strict personal protective measures and systematic disinfection. PCR technology plays a significant role in detecting SARS-CoV-2 RNA.

### 2.2. Nucleic Acid Detection Based on Isothermal Amplification Technology

Isothermal amplification technology has been developed for more than 20 years as an alternative to a polymerase chain reaction. The main advantage of isothermal amplification is that it does not require expensive thermal cycling laboratory equipment [30]. Researchers have developed new methods based on the main shortcomings of each technology, hoping to reduce the negative impact of defects and achieve rapid, accurate, and low-cost detection of SARS-CoV-2 [31].

**Figure 2:**
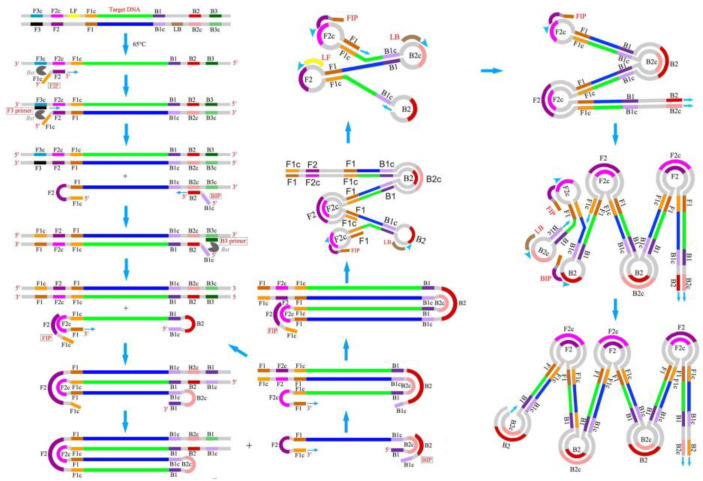
Schematic of LAMP [33]. (Copyright 2016 Li, Xiong, Liu, Liang and Zhou).

Loop-mediated isothermal amplification (LAMP) technology is one of the nucleic acid isothermal amplification technologies, which has the advantages of high sensitivity, fast response speed, simple operation, and easy observation of results. When the LAMP reaction starts, the forward inner primer hybridizes with the original reverse target sequence and the synthesis of the new forward strand starts from the 3′ end flanked by the forward inner primer. Then, the forward outer primer hybridizes again with the same original reverse target sequence and the synthesis of this new forward strand continues until the enzyme finds the 5′ end of the first strand created with the use of the inner primer. This separated strand creates a self-hybridizing loop at one end owing to the complementarity of the reverse sequence from the inner primer to the target sequence, thus creating a dumbbell-like DNA structure. The forward inner primer then hybridizes to the chain loop formed in the initial step and promotes strand displacement to generate a new strand. Subsequently, self-primed strand displacement DNA synthesis produces two products, a complementary strand and a strand with a double elongated stem that is as long as the original strand and the loop at the opposite site. Both strands are then used as templates for the reverse-primed strand displacement synthesis in the subsequent elongation and recycling steps (Figure 2). Finally, stem-loop DNA of different lengths and cauliflower-like structures with multiple loops are generated [32].

Adding fluorescent dyes (such as SYBR Green I) or other dyes (such as hydroxynaphthol blue) to the LAMP system is capable of achieving visual detection [34,35,36,37]. The sensitivity of LAMP to inhibitors in the sample is lower than that of general PCR, which also means that LAMP can cope with more complex and diverse detection environments. Reverse transcriptase loop-mediated amplification (RT-LAMP) needs to convert SARS-CoV-2 RNA into cDNA and then amplify it with 4–6 primers. Adding high-fidelity DNA polymerase to the LAMP system will improve sensitivity and speed [38]. At the time of the outbreak, Renfei et al. successfully detected the RdRp gene fragment of SARS-CoV-2 using this principle and RT-LAMP technology. The reaction results can be obtained in a few minutes. They tested for 17 common human respiratory diseases, proving that this test is reliable [39]. In the face of weakly positive samples, the detection technology needs to improve the detection sensitivity. The effect of various additives on the detection performance of LAMP was tested by Mohammed’s group. They found that addition of 1 mg/mL bovine serum albumin (BSA) could increase the sensitivity of assay up to 10 copies of target sequence. After adding BSA to the LAMP system, the RdRp nucleic acid of SARS-CoV-2 in clinical specimens of COVID-19 will be successfully detected within 20 min. In addition, this method can also detect SARS-CoV-2 in sewage collected locally [40]. Another researcher developed a way to improve the sensitivity of RT-LAMP by adding 40 mM guanidine hydrochloride (pH 8.0) to the LAMP reaction. They selected a dual-target gene for SARS-CoV-2 (Spike (S) protein and RNA-dependent RNA polymerase (RdRP)) for detection, increasing sensitivity to 25–50 copies per reaction [41].

In conclusion, adding appropriate concentrations of BSA and guanidine hydrochloride to the detection reagent can improve the sensitivity of LAMP.

**Figure 3:**
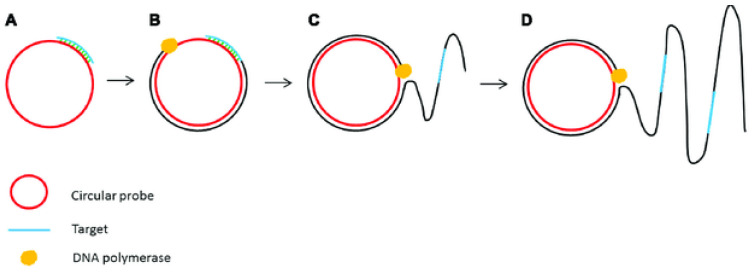
Schematic outline of rolling circle amplification (RCA). (**A**) A primer complementary to a region of a circular probe anneals to the circular template. (**B**) DNA polymerase initiates the DNA synthesis. (**C**) Strand displacement allows the continuation of DNA synthesis along the circular template. (**D**) DNA synthesis continues to generate a long ssDNA product [42]. (Copyright 2017 Lau and Botella).

RCA is a DNA amplification technique established in 1998 that can be used to amplify large circular DNA templates and can occur at constant temperature.This technology is a nucleic acid amplification technology based on the rolling circle replication method of circular pathogenic microorganism DNA molecules in nature.The polymerases used in the experiment now mainly include Phi 29, Bst, Phage T7, etc. These enzymes have strong persistence and strand displacement ability, which can meet the requirements of the RCA amplification mechanism. The polymerase can continuously extend a single strand of circular DNA template and return to the origin after completing a circle of replication. The displacement activity between nucleic acid chains makes the newly synthesized nucleic acid chain further replace the previous old chain as the polymerization precursor, so the extension process can be further circulated (Figure 3) [42].

**Figure 4:**
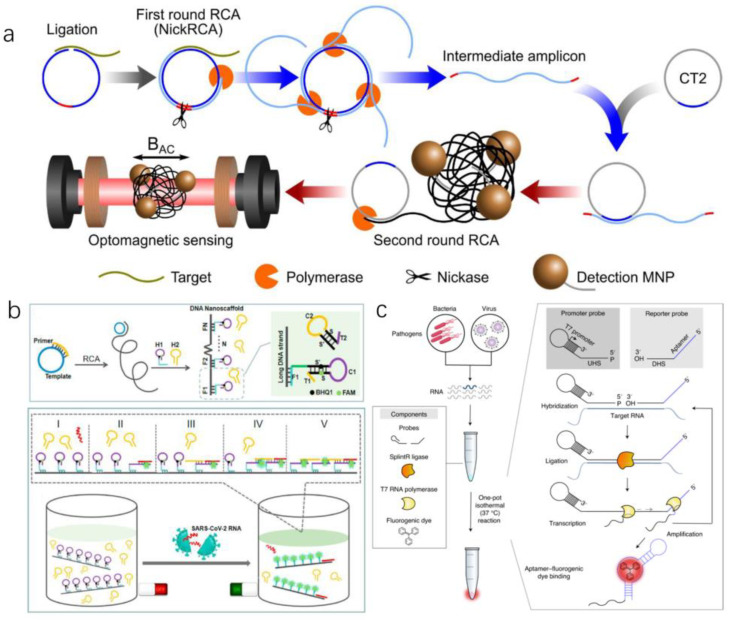
(**a**) Schematic illustration of homogeneous circle-to-circle amplification [43]. (**b**) A DNA nanoscaffold hybrid chain reaction (DNHCR)-based method for the detection of SARS-CoV-2 RNA [44]. (**c**) Schematic of SENSR, a one-pot isothermal reaction cascade for the rapid detection of RNA [48]. (Copyright © 2020 Elsevier B.V. All rights reserved.) (Copyright © 2020 Elsevier B.V. All rights reserved.) (Copyright Copyright © 2020, The Author(s), under exclusive licence to Springer Nature Limited).

Circle-to-circle amplification (C2CA) is an exceptional and accurate cascade nucleic acid amplification approach that unites two rolling circle amplification (RCA) loops in a one-step amplification reaction (Figure 4a). The second round of RCA produces amplicon coils that anneal to detection probes grafted onto MNPs, resulting in MNP assembly that can be detected in real-time using an optomagnetic sensor. The method achieved a subfemtomolar level detection limit [43]. In addition to this, isothermal amplification reactions can be combined with cascade amplification reactions to detect SARS-CoV-2. Jiao et al. reported a nucleic acid detection strategy based on rolling loop amplification and a DNA nanoscaffold hybridization chain reaction (DNHCR) for SARS-CoV-2 RNA rapid detection (Figure 4b). In this way, long-stranded DNA and a self-quenching probe (H1) form a DNA nanoscaffold. Then, the SARS-CoV-2 RNA will initiate the hybridization of H1 and free H2 DNA probes along the nanoscaffold, and an illuminated DNA nanostring is obtained immediately. The method can detect the target in short (within 10 min) and under mild conditions (15–35 °C) [44]. In addition, one-pot, ligation-dependent isothermal cascade reactions (SENSR) have been developed by Woo’s research group (Figure 4c). The RNA aptamer [45,46,47] binds to the fluorescent dye and fluoresces only when the target RNA is present in the sample. Unbound fluorescent dyes dissipate their energy in the form of molecular vibration and heat, which prevents the increase of fluorescence [48].

Most isothermal detection techniques could complete detection in less than 1 h. In contrast to SENSR, both DNHCR and C2CA require a preamplification step for detection. Many isothermal detection techniques have reduced LOD to varying degrees. SENSR reduces the LOD to 0.1 aM, but the sensitivity is low compared to other detection techniques. In conclusion, rapid and easy isothermal detection techniques have the potential to replace traditional detection techniques (Table 2).

### 2.3. Nucleic Acid Detection Based on RNA-Guided CRISPR–Cas System

**Figure 5:**
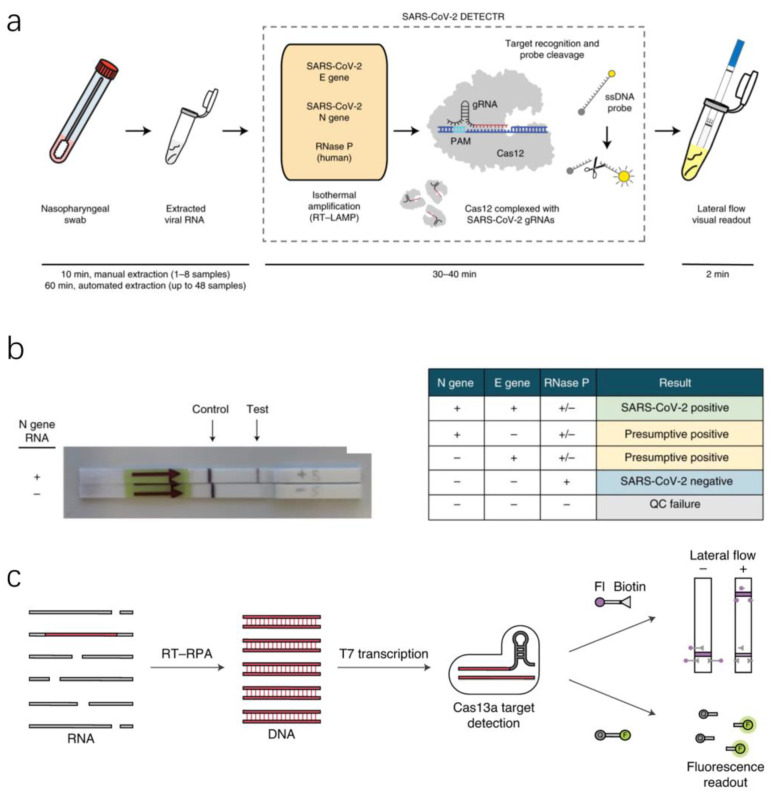
A CRISPR–Cas-based assay for detection of SARS-CoV-2. (**a**) Conventional RNA extraction can be used as an input to DETECTR, which is visualized by a fluorescent reader or lateral flow strip [52]. (**b**) Lateral flow strip assay readout. A positive result requires the detection of at least one of the two SARS-CoV-2 viral gene targets. QC, quality control [52]. (**c**) SHERLOCK detection of SARS-CoV-2 RNA [53]. (Copyright © 2020, The Author(s), under exclusive licence to Springer Nature America, Inc.) (Copyright © 2020, The Author(s), under exclusive licence to Springer Nature Limited).

CRISPR–Cas systems, especially CRISPR–Cas12a and CRISPR–Cas13a, characterized by their sensitivity, specificity, high base resolution, and programmability upon nucleic acid recognition, have been repurposed for molecular diagnostics, surging a new path forward in biosensing. As the core of some robust diagnostic tools, they are revolutionizing the way of detection [16]. In 2017, Zhang Feng’s team developed a diagnostic tool based on the CRISPR–Cas13a system called Specific High-Sensitivity Enzymatic Reporter Unlocking (SHERLOCK), which can perform ultrasensitive and specific identification of DNA or RNA in clinical samples [49]. Based on the CRISPR–Cas system, Ding et al. proposed the All-In-One Dual CRISPR–Cas12a (AIOD-CRISPR) detection system. Unlike the regular CRISPR–Cas12a system, dual crRNA was introduced in this study to efficiently initiate dual CRISPR-based nucleic acid detection [50]. In addition, the visual response of CRISPR–Cas12 detection used the FAM-biotin reporter and a lateral flow strip (Figure 5a,b) [51]. Uncleaved reporter molecules are captured at the first detection line (control line), whereas indiscriminate Cas12 cleavage activity generates a signal at the second detection line (test line) [52]. Patchsung et al. amplified the SARS-CoV-2 target RNA region isothermally into DNA by reverse transcription recombinase polymerase amplification (RT-RPA) and then converted it into RNA by T7 polymerase transcription (Figure 5c). Cas13a-crRNA complex binds to RNA targets and activates Cas13a, which is capable of cleaving RNA reporters [53].

In summary, CRISPR–Cas detection technology generally amplifies nucleic acids and adds them to the detection system afterward. From Table 3, it can be seen that the sensitivity of the CRISPR–Cas system is comparable to that of PCR, the detection speed is faster than that of PCR, and the overall performance is better than that of PCR. The CRISPR–Cas system initiated by dual crRNA has the best sensitivity and the fastest detection speed. However, the cost of this method in practical application is much higher than PCR, and it is not suitable for large-scale detection.

## 3. Ultrasensitive Microfluidic for SARS-CoV-2 Detection

SARS-CoV-2 is highly contagious and has spread to more than 210 countries worldwide [54,55]. Each country needs to quickly detect the new coronavirus (SARS-CoV-2) in the population, but this requires many biological reagents, which is a massive challenge for each country [56,57,58]. The above reports report a variety of exciting and ingenious off-chip detection techniques, all of which have great potential. Currently, they are only suitable for laboratory testing, which means that they require professional operators and specialized equipment, which is not enough to play a positive role in mass screening. If they combine with microfluidics to address the limitations of current methods, detection techniques could become more practical. First of all, it can realize integrated miniaturization and automation and concentrate multiple steps of sample detection on a small chip. Second, it can achieve high-throughput detection and detect various items in parallel on the same sample as needed. Compared with conventional item-by-item detection, it shortens detection time, improves detection efficiency, and avoids cross-contamination between reagents. The amount of reagents used in the third microfluidic chip is far lower than that of conventional reagents, which significantly reduces the consumption of reagents. Fourth, it usually only takes microliters or even nanoliters of sample volumes [59,60,61]. Microfluidics has received much attention worldwide due to its multiple advantages [62].

### 3.1. Microfluidic Detection Based on PCR Technology

For PCR reactions, the microscale structure in the microfluidic devices (meaning a larger surface area to volume ratio) can transfer heat faster [63], thereby shortening the thermal cycle time in qRT-PCR analysis and saving energy consumption. A reasonably designed microfluidic device can seal the reaction system, reducing the aerosol contamination caused by frequent lid opening, which is a complex problem in nucleic acid amplification [64,65]. These devices will detect the nucleic acid of the SARS-CoV-2 conveniently, economically, and efficiently. At present, many groups have developed equipment for detecting SARS-CoV-2 based on microfluidic technology. It has long been recognized that miniaturization and automated handling of fluid samples are key to the successful commercialization of lab-on-a-chip systems [66].

**Figure 6:**
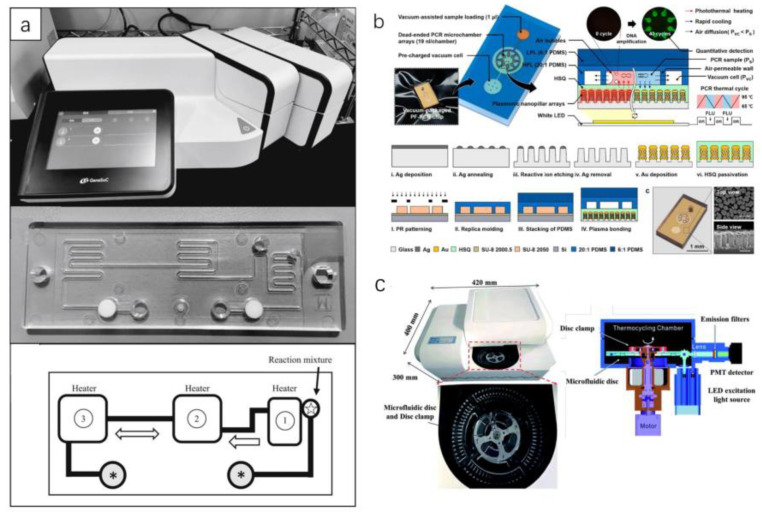
Detection of SARS-CoV-2 by PCR on microfluidic. (**a**) Schematic diagram of space domain PCR equipment, microfluidic chip, and liquid flow [17]. (**b**) Vacuum-assisted nanoplasmonic on-chip polymerase chain reaction [67]. (**c**) Thermocycling and optical detection in microfluidic devices, microfluidic chips, and devices [63]. (Copyright © 2020 The Healthcare Infection Society. Published by Elsevier Ltd. All rights reserved.) (Copyright © The Royal Society of Chemistry 2020) (Copyright © 2021 The Authors. Published by American Chemical Society).

A microfluidic detection device was reported by Sakai et al. (Figure 6a). The device comprises three heaters, one for reverse transcription (RT) reactions and the other two for thermal cycling. There are two microblowers at both ends of the fluid channel, and the PCR solution shuttles through the channel at high speed using the wind. The device could also monitor the real-time fluorescence intensity of the PCR solution [17]. The detection device may be performed outside the laboratory. However, the sampling procedure and time may affect the detection result.

The PCR technology in the microfluidics introduced above belongs to the spatial domain PCR. Spatial domain PCR is the realization of the thermal cycling of the PCR reaction solution in the microfluidic chip through the transfer of the spatial position. Another type of PCR technology in microfluidics is time-domain PCR. Time-domain PCR is the thermal cycle of the PCR reaction solution in the microfluidic chip by heating and cooling. Byoung-Hoon et al. report a microfluidic chip for time-domain PCR, using nanoplasmonic and vacuum-assisted microfluidics technology for rapid and quantitative molecular diagnosis (Figure 6b). Using the strong light of a white light-emitting diode, glass nanopillar arrays with Au nanoislands will produce ultrafast nanoplasmonic heating; in addition, rapid cooling can be achieved due to the large surface volume ratio of nanopillars [67]. The light power of the LED provides the energy required for the PCR reaction in this microfluidic chip.

Centrifugal microfluidic technology is an essential branch of microfluidic technology. It is different from mechanical movements such as micropump and magnetic bead drive. It does not need to connect to any other external interfaces and realize the integration and simplicity of the analysis system. Due to the centrifugal force field, the centrifuge chip easily removes any air bubbles that might interfere with the assay, and the length of the channel and rotational speed are adjusted to vary the centrifugal force. The centrifugal chips drive tens or hundreds of individual microfluidics with a simple spindle motor. The independent structural unit performs molecular diagnosis and detection analysis on the sample. More and more studies have shown that the combination of centrifugal microfluidic chips and nucleic acid isothermal amplification technology has the characteristics of miniaturization, integration, and automation.

Minghui et al. designed a centrifugal microfluidic chip (Figure 6c). The chip delivers detection reagents to the reaction chamber by adjusting the rotation speed. It then drives the PCR reaction mixture to different temperature zones repeatedly with the support of a two-way turntable for heating [63]. The device allows real-time monitoring of the fluorescence intensity in the microreaction chamber for quantitative detection of SARS-CoV-2 RNA.

From Table 4, we know that the detection chip proposed by Byoung-Hoon’s team can quickly perform PCR reactions within 6 min, and the detection speed is faster than the other two chips. The reason is that they take full advantage of the high specific surface area of microfluidic chips. Cleverly, they used light energy to power the PCR reaction. Minghui’s team’s centrifugal microfluidic chip increases the speed of sample addition, which aids large-scale screening. In conclusion, microfluidic chips can speed up nucleic acid detection.

### 3.2. Microfluidic Detection Based on Isothermal Amplification Technology

Using isothermal amplification technology to detect nucleic acids in microfluidic devices has become a trend. Compared with PCR technology, isothermal amplification technology has obvious advantages. It requires only a single temperature to perform nucleic acid amplification. This simple requirement simplifies the hardware conditions for nucleic acid detection.

#### 3.2.1. LAMP in Microfluidics

**Figure 7:**
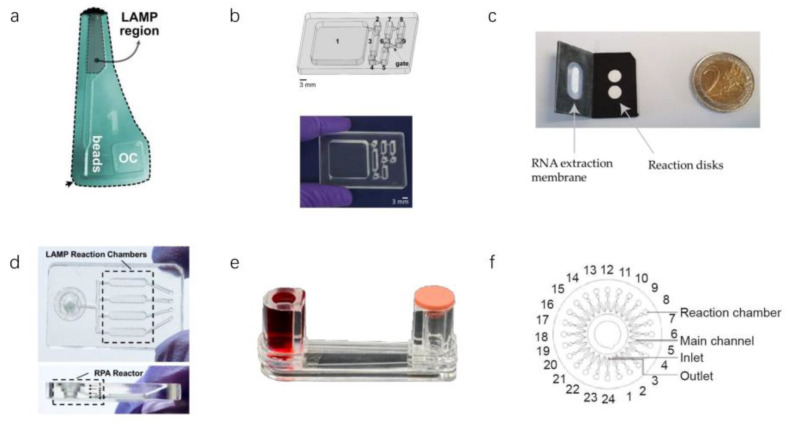
Several essential microfluidic chips for detecting SARS-CoV-2 using isothermal amplification. (**a**) PMMA microfluidic chip based on accurate LAMP detection [68]. (**b**) Microfluidic chip and liquid flow diagram of IFAST RT-LAMP [69]. (**c**) Paper-based microfluidic chip composed of polypropylene (black) and glass fiber (in white) [70]. (**d**) LAMP reaction region and RPA reaction region in microfluidic chip [18]. (**e**) Microfluidic detection device based on DNA gel [72]. (**f**) Schematic diagram of microfluidic chip [14]. (Copyright © The Royal Society of Chemistry 2021) (Copyright © 2021 Elsevier B.V. All rights reserved.) (Copyright © 2021 Garneret et al.) (Copyright © 2021 Elsevier B.V. All rights reserved.) (Copyright © 1969, Elsevier) (Copyright © 2020 THE AUTHORS. Published by Elsevier LTD on behalf of Chinese Academy of Engineering and Higher Education Press Limited Company).

qLAMP is a rapid isothermal quantitative detection technique, but the numerous and complex LAMP primers in qLAMP reactions tend to form primer-dimers, resulting in nonspecific fluorescent signals. To eliminate this deleterious effect, Soares et al. reported a centrifugal microfluidic chip containing the ligand N-benzyl N-methyl ethanolamine (Figure 7a) [68]. The chip can filter out the remaining primers after the LAMP reaction so that the fluorescence value after the qLAMP response is more accurate. However, unstable detection limits and complicated operating procedures are unsatisfactory. The naked eye also can directly detect the reaction results of LAMP. A detection chip based on “immiscible filtration” nucleic acid extraction was reported by Rodriguez Mateos’s research group (Figure 7b). They used magnetic-bead-purified nucleic acids as samples for the LAMP reaction. After the reaction, inspectors determines the test results by observing the color of the liquid in the reaction chamber [69]. Due to this method requiring manual nucleic acid purification by professionals, the popularization of this technique has encountered difficulties. If nucleic acids can be automatically purified in other way, this will significantly reduce operator requirements and thus improve detection efficiency. LAMP even can detect viruses in resource-constrained environments. Garner et al. reported a paper-based microfluidic chip for detecting SARS-CoV-2 (Figure 7c). The chip can extract RNA and can also perform the LAMP reaction. The cost of this portable assay device is around $2–4, making the chip suitable for dissemination both in developed countries and in countries with limited resources [70]. This paper-based microfluidic detection chip is indeed convenient and inexpensive. However, this detection chip requires extensive and complicated manual operations, which is unsatisfactory. LAMP technology also can combine with other isothermal amplification technologies in microfluidic chips. Compared with only one amplification in a microfluidic chip, two-stage amplification in the chip can detect a lower concentration of SARS-CoV-2 RNA. Yin et al. reported a chip for the detection of SARS-CoV-2 (Figure 7d). The template is amplified by RPA in the first reaction zone, and the reaction products are coenhanced in the second reaction zone by LAMP [18]. They realized smart, connected, on-site detection with a reporting framework embedded in a portable detection platform, which exhibited potential for rapid spatiotemporal epidemiologic data collection regarding the environmental dynamics, transmission, and persistence of infectious diseases. Although this detection method improves sensitivity, its operation is complicated. First, the form contains two isothermal amplification techniques, so two different temperatures are required for the reaction, which requires more complex heating equipment. Second, adding samples takes a long time and involves multiple manual operations, which is not ideal for rapid on-site detection. Third, the two isothermal amplification techniques require more biochemical reagents, making it difficult to reduce the product’s price. Perhaps they could choose a smaller reaction chamber, saving costs by using fewer biochemical reagents. A microfluidic detection device consisting of multiple effect transistors was reported by Rodriguez Manzano et al. The device is combined with RT-LAMP technology to detect nucleic acids by monitoring the pH changes during the LAMP reaction [71]. The device has the advantages of a meager detection limit, short detection time, and small size. However, the device still has many shortcomings. First, the reagents for the LAMP reaction still need to be compounded externally and cannot be integrated. Second, the device can only detect one sample at a time and cannot add a blank control, which increases the risk of false negatives in the experimental results.

In conclusion, LAMP technology and microfluidic technology are suitable for nucleic acid detection. First, the reaction result of LAMP can be judged in various ways (color change, fluorescence, and pH value). Second, LAMP can be applied to paper-based microfluidics to reduce detection costs. Finally, LAMP can combine with other biotechnologies to reduce LOD in microfluidics.

#### 3.2.2. Other Isothermal Amplification Techniques in Microfluidics

Researchers have proposed various detection techniques to reduce LOD as much as possible. Kim et al. reported a consumable test similar to a U-tube with a sample chamber and a waste chamber with a rubber cap on the other side, linked by a glass tube (Figure 7e). The detection device has a low LOD (0.7 aM) [72]. Xing et al. reported an isothermal amplification analyzer (Figure 7f). The system sends samples to 16 reaction chambers through centrifugal force and produces test results after 35 min of reaction [14].

RPA and nucleic acid sequence-based amplification (NASBA) are also efficient and sensitive isothermal amplification techniques that can replace PCR and LAMP for virus detection in centrifuged microchips. From Table 5, we found that they have sensitivity comparable to LAMP. RPA has a satisfactory sensitivity and response speed. However, most reported microfluidic detection chips require off-chip preprocessing. All LAMP-based detection chips can obtain detection results within 60 min. In conclusion, isothermal technology can detect viruses easily and rapidly in microfluidic chips.

### 3.3. Microfluidic Detection Based on CRISPR–Cas Biological Detection Technology

CRISPR–Cas biology provides a new method for fast and efficient detection of pathogens. The CRISPR–Cas diagnostic process has advantages such as rapidity and specificity, but it requires prepurified nucleic acids, many reagents, and several manual steps. These factors hinder its development and application in a low-resource environment. The researchers have combined microfluidics and CRISPR–Cas methods to address the limitations of current CRISPR–Cas detection methods.

**Figure 8:**
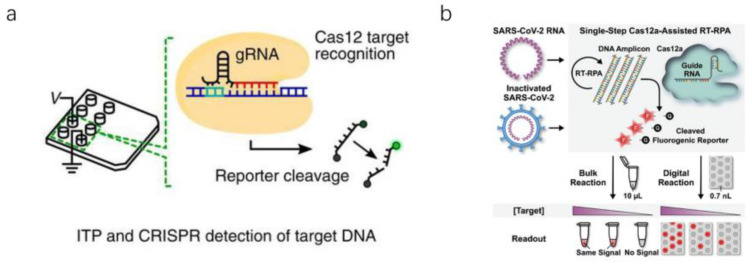
Several essential microfluidic chips for detecting SARS-CoV-2 using CRISPR–Cas. (**a**) Electric-field-mediated microfluidic detection device [19]. (**b**) Schematic diagram of digital CRISPR CAS [73]. (Copyright © 2020 the Author(s). Published by PNAS.) (Copyright © 2021 The Authors. Advanced Science published by Wiley-VCH GmbH).

As is known to all, whether samples and detection reagents are throughly mixed is one of the key factors affecting the success of biological detection. Ramachandran et al. reported an electric microfluidic device based on isotachophoresis (ITP) (Figure 6a). With the ITP technique, various detection reagents can be effectively mixed, accelerating the reaction rate [19]. Park et al. reported a digitally enhanced CRISPR–Cas virus detection application for SARS-CoV-2 detection (Figure 8b) [73]. It is one of the fastest and most sensitive CRISPR–Cas-assisted SARS-CoV-2 detection methods. However, we see several routes for advancing digitization-enhanced CRISPR/Cas-assisted one-pot virus detection (deCOViD). First, the speed and sensitivity of deCOViD could be improved by optimizing the ratio of each component in the reagent. Second, the fluorescence detection and heating devices can be miniaturized and assembled into portable apparatus.

In this article, we discuss many microfluidic detection techniques and devices that are portable, low-cost, and can perform detection quickly. Each detection method has different highlights. For example, U-tube-based microfluidic devices can detect viruses at low concentrations [72]. The detection technology based on the centrifugal microfluidic chip can realize high-throughput detection and improve detection efficiency [14,63]. The detection technology based on paper-based microfluidics can reduce the detection cost [70]. The electronics-based microfluidic device is tiny and portable for home self-inspection [71]. However, these chips and devices also have many limitations. For example, some detection methods require the configuration of reagents and sampling outside the equipment, and these operations still require complicated manual processes [14,68,71,72]. Therefore, researchers should further develop highly automated microfluidic devices. At present, because some microfluidic chips are expensive to manufacture, this increases the cost of detection. Because the nucleic acid in the cavity is hard to remove, the centrifugal microfluidic chip cannot be reused after the reaction. New structures are required [14,63,68]. Moreover, chips and devices are mostly tested in labs, not in the real application scenarios, so the repeatability and durability of the equipment need to be improved and tested.

## 4. Conclusions

### 4.1. Summary on Traditional and Microfluidic Experiments

This article discussed conventional methods with advantages and disadvantages and microfluidic methods with advantages and disadvantages for SARS-CoV-2 RNA detection [74,75]. Compared with microfluidic methods, traditional techniques generally have higher reliability. However, the shortcomings are also evident. Compared with microfluidic chip detection technology, conventional methods require a great amount of biological reagents (enzymes, primers, and buffers). Traditional methods require professional operations, rigorous operating procedures, and laboratories with sophisticated instruments. At the same time, the samples taken from the population need further processing before they are used for testing by conventional methods. The researchers designed the structure and function of the microfluidic chips, integrated purified nucleic acid, amplified nucleic acid, and detection products into one microchip, and realized the rapid detection of “sample-to-answer” [76,77,78]. Microfluidic chip technology has the advantages of low operator requirements and low detection cost, and it is expected to replace the traditional detection technology for large-scale screening of the population.

### 4.2. Summary on Nucleic Acid Detection Technology

From another perspective, we discussed PCR technology, isothermal amplification technology, and CRISPR–Cas biotechnology for SARS-CoV-2 RNA detection. PCR is a relatively mature biological detection technology. Most countries and regions use this technology for SARS-CoV-2 RNA detection. Judging from Table 1 and Table 4, on-chip and off-chip PCR technologies require thermal cycle amplification, and there is a heating and cooling process in thermal cycling, which involves much time. The spatial domain PCR technique will improve this drawback to some extent. PCR assays with disadvantages need complex thermal cycling equipment and high cleanliness requirements for operation. Besides, the reaction process of isothermal amplification technology is always maintained at a constant temperature, and nucleic acid is also rapidly amplified [79,80,81]. Compared with PCR detection technology, isothermal amplification technology improves the detection speed. Although there have been many reports on detecting SARS-CoV-2, most are still in the laboratory research stage. Regardless of whether it is on-chip or off-chip isothermal amplification technology, relevant standards have not yet been established. The main reason is that there are still some problems in applying these methods [82]. From Table 5, the LAMP is the most used isothermal amplification technique, both on-chip and off-chip, which produces a more significant number of specific products with high sensitivity and specificity. Compared with other isothermal amplification techniques, the reaction results of LAMP can be judged by visual, electrophoretic, and turbidimetric methods, which is why most researchers choose this technique to detect SARS-CoV-2. There are some shortcomings in isothermal amplification technology. LAMP, RCA, NASBA, and RPA require highly demanding primers (specific primers). Second, isothermal amplification products are mostly multicopy long chains formed by the same fragment. The concentration of the amplified target fragments of the amplified products that exist due to sol contamination has increased dozens of times or even thousands of times, so false positives are elementary to produce. Therefore, the experiment must strictly distinguish the solution preparation area, the sample processing area, and the detection area. Therefore, there is still much room for improvement in nucleic acid isothermal amplification technology. Compared with the traditional PCR technology, NAT based on the CRISPR–Cas system has higher sensitivity, better specificity, convenience, and low cost. Therefore, it has become a dazzling star in biological detection. However, the current NAT based on the CRISPR–Cas system has some shortcomings, which are mainly reflected in the following aspects. CRISPR-based nucleic acid detection technology generally requires RNA fluorescent reporter probes, but the RNA is quickly degraded, leading to false-positive results. Due to the low sensitivity of Cas13 detection alone, CRISPR-based nucleic acid detection technology generally requires two reaction steps. The first step is the amplification reaction, and the second step is to add the amplified sample to a tube containing reaction reagents such as CAS protein for the detection reaction, which not only increases the complexity of the detection process but may also contaminate the sample during the transfer step. Therefore, there is a need to further develop simpler assays that allow the amplification of RNA or DNA and the chemical reaction of Cas protein detection to be performed in the same tube, or to explore more novel Cas and establish simpler and more efficient amplification-free CRISPR–Cas assays.

## 5. Future Perspectives

At the beginning of the pandemic, many commercial products based on microfluidics were developed [83,84,85,86] and gained widespread popularity. It is a sign of the technology and the business models maturing [87]. In recent years, microfluidic devices have been used to diagnose a variety of viruses and clinical applications and have been generally recognized by academia and industry [88]. However, microfluidic technology has not brought a huge impact on traditional in vitro diagnostic products as expected. Therefore, the industrialization of microfluidic technology and the introduction of real practical microfluidic products should become one of the main goals in this field.

Following the advent of COVID-19, our society has encountered unprecedented health and economic situation. A second wave is starting to strike some countries, and healthcare organizations are now testing many more people than several months ago. However, this is not a one-shot rise in revenue that will come back to normal soon. Once the pandemic is over, these instruments will remain in place and be used for other tests that these companies offer. It will take their consumables sales to a higher level than before the pandemic. Moreover, governments and society have realized the importance of such diagnostic tests. They will surely support their continued development to be ready in case of another future pandemic. Therefore, the diagnostics industry may make the best of this bad situation and turn it into a real springboard. Overall, the future detection market belongs to microfluidics.

## Figures and Tables

**Table 1:** The data and applications of off-chip PCR detection technology were summarized.

Sample Type	Sensitivity	Target Genes	Application Scenario	Assay Time	Reference
Oropharyngeal swabs (OPS)	97.4%	-	Lack of nucleic acid extraction reagent	PCR45 cycles	[25]
Oropharyngeal swabs (OPS)	94%	N	Laboratory testing	PCR40 cycles	[23]
Saliva	95%	RdRP	Throat swabs and protective equipment are in short supply	PCR30 cycles	[26]
Wastewater	-	E	Outdoor water source	40 cycles	[28]
Air	-	-	hospital	45 cycles	[29]

**Table 2:** The data and applications of off-chip isothermal amplification detection technology were summarized.

Sample Type	Lower Detection Limit	Sensitivity	Target Genes	Application Scenario	Assay Time	Reference
Clinical sample	-	100%	RdRP	Isothermal heating instrument	40 min	[39]
Synthetic DNA	0.4 fM	100%	RdRP	Laboratory	100 min	[43]
Synthetic DNA	0.96 pM	-	-	Fluorescence detector	10 min	[44]
Clinical sample, nasopharyngeal swabs (NPS)	0.1 aM	95%	RdRp	Fluorescence detector	30–50 min	[48]
Sewage and clinical samples	10 copies	93%	RdRp	Visual observation	20 min	[40]
Nasopharyngeal swabs (NPS)	25–50 copies/Reaction	95.8%	RdRp, S	Fluorescence detector	30 min	[41]

**Table 3:** The data and applications of off-chip CRISPR–Cas detection technology are summarized.

Sample Type	Lower Detection Limit	Sensitivity	Target Genes	Application Scenario	Assay Time	Reference
Clinical sample	-	100%	N	Hand warmer	20 min	[50]
Oropharyngeal swabs (OPS), nasopharyngeal swabs (NPS)	-	95%	E+N	Limited medical resources	40 min	[52]
Oropharyngeal swabs (OPS), nasopharyngeal swabs (NPS)	42/Reaction	96%	N, Orf1ab	Limited medical resources	1 h	[53]

**Table 4:** The data and applications of on-chip PCR detection technology were summarized.

Sample Type	Lower Detection Limit	Sensitivity	Target Genes	Application Scenario	Assay Time	Reference
Oropharyngeal swabs (OPS), nasopharyngeal swabs (NPS)	10 copies/μL	100%	N	Centrifugal microfluidic detection instrument	1.5 h	[63]
Nasopharyngeal swabs (NPS)	10 copies/20 μL	92%	N	Centrifugal microfluidic detection instrument	95 min	[17]
Synthesis Plasmid	-	-	E	Lighting	306 s	[67]

**Table 5:** The characteristics and data of on-chip isothermal amplification technology are summarized and compared.

Pretreatment (On-Chip and Off-Chip)	Sample Motion (Centrifugation, Capillary Force)	Lower Detection Limit	Target Genes	Test Method	Assay Time	Reference
On-chip	Magnetic force	470 copies/mL	ORF1a, N	RT-LAMP	30–45 min	[69]
Off-chip	-	10 copies/reaction	N	RT-LAMP	20 min	[71]
On-chip	Fold	1 copy/μL	ORF1ab	RT-LAMP	20–60 min	[70]
On-chip	Propulsive force	100 (GE)/mL	E, N ORF1a	RT-LAMP	60 min	[18]
Off-chip	-	0.7 aM	-	RPA	15 min	[72]
Off-chip	Centrifugal force	50 copies/μL	S, N	LAMP	90 min	[68]
Off-chip	Centrifugal force	100 and 1000 copies/10 μL	ORF1ab	NASBA	60 min	[14]

## Data Availability

Data sharing not applicable. No new data were created or analyzed in this study.

## References

[1] Wu F., Zhao S., Yu B., Chen Y.M., Wang W., Song Z.G., Hu Y., Tao Z.W., Tian J.H., Pei Y.Y. (2020). A new coronavirus associated with human respiratory disease in China. Nature.

[2] Wang D., Hu B., Hu C., Zhu F., Liu X., Zhang J., Wang B., Xiang H., Cheng Z., Xiong Y. (2020). Clinical Characteristics of 138 Hospitalized Patients with 2019 Novel Coronavirus-Infected Pneumonia in Wuhan, China. JAMA J. Am. Med. Assoc..

[3] Cevik M., Kuppalli K., Kindrachuk J., Peiris M. (2020). Virology, transmission, and pathogenesis of SARS-CoV-2. BMJ.

[4] Chen N., Zhou M., Dong X., Qu J., Gong F., Han Y., Qiu Y., Wang J., Liu Y., Wei Y. (2020). Epidemiological and clinical characteristics of 99 cases of 2019 novel coronavirus pneumonia in Wuhan, China: A descriptive study. Lancet.

[5] Zhao J., Yuan Q., Wang H., Liu W., Liao X., Su Y., Wang X., Yuan J., Li T., Li J. (2020). Antibody Responses to SARS-CoV-2 in Patients with Novel Coronavirus Disease 2019. Clin. Infect. Dis..

[6] Long Q.X., Liu B.Z., Deng H.J., Wu G.C., Deng K., Chen Y.K., Liao P., Qiu J.F., Lin Y., Cai X.F. (2020). Antibody responses to SARS-CoV-2 in patients with COVID-19. Nat. Med..

[7] Lu R., Zhao X., Li J., Niu P., Yang B., Wu H., Wang W., Song H., Huang B., Zhu N. (2020). Genomic characterisation and epidemiology of 2019 novel coronavirus: Implications for virus origins and receptor binding. Lancet.

[8] Ren X., Liu Y., Chen H., Liu W., Guo Z., Zhang Y., Chen C., Zhou J., Xiao Q., Jiang G.-M. (2020). Application and optimization of RT-PCR in diagnosis of SARS-CoV-2 infection. SSRN Electron. J..

[9] Zhou P., Yang X.L., Wang X.G., Hu B., Zhang L., Zhang W., Si H.R., Zhu Y., Li B., Huang C.L. (2020). A pneumonia outbreak associated with a new coronavirus of probable bat origin. Nature.

[10] Wang J., Wang Z. (2020). Strengths, weaknesses, opportunities and threats (Swot) analysis of china’s prevention and control strategy for the COVID-19 epidemic. Int. J. Environ. Res. Public Health.

[11] Berkenbrock J.A., Grecco-Machado R., Achenbach S. (2020). Arsenal of microfluidic testing devices may combat COVID-19 pandemic. MRS Bull..

[12] Yang L., Yamamoto T. (2016). Quantification of virus particles using nanopore-based resistive-pulse sensing techniques. Front. Microbiol..

[13] Zhu H., Fohlerová Z., Pekárek J., Basova E., Neužil P. (2020). Recent advances in lab-on-a-chip technologies for viral diagnosis. Biosens. Bioelectron..

[14] Xing W., Liu Y., Wang H., Li S., Lin Y., Chen L., Zhao Y., Chao S., Huang X., Ge S. (2020). A High-Throughput, Multi-Index Isothermal Amplification Platform for Rapid Detection of 19 Types of Common Respiratory Viruses Including SARS-CoV-2. Engineering.

[15] Shu B., Zhang C., Xing D. (2014). Segmented continuous-flow multiplex polymerase chain reaction microfluidics for high-throughput and rapid foodborne pathogen detection. Anal. Chim. Acta.

[16] Yin L., Man S., Ye S., Liu G., Ma L. (2021). CRISPR-Cas based virus detection: Recent advances and perspectives. Biosens. Bioelectron..

[17] Sakai J., Tarumoto N., Orihara Y., Kawamura R., Kodana M., Matsuzaki N., Matsumura R., Ogane K., Kawamura T., Takeuchi S. (2020). Evaluation of a high-speed but low-throughput RT-qPCR system for detection of SARS-CoV-2. J. Hosp. Infect..

[18] Yin K., Ding X., Xu Z., Li Z., Wang X., Zhao H., Otis C., Li B., Liu C. (2021). Multiplexed colorimetric detection of SARS-CoV-2 and other pathogens in wastewater on a 3D printed integrated microfluidic chip. Sens. Actuators B Chem..

[19] Ramachandran A., Huyke D.A., Sharma E., Sahoo M.K., Huang C., Banaei N., Pinsky B.A., Santiago J.G. (2020). Electric field-driven microfluidics for rapid CRISPR-based diagnostics and its application to detection of SARS-CoV-2. Proc. Natl. Acad. Sci. USA.

[20] Chen L., Liu W., Zhang Q., Xu K., Ye G., Wu W., Sun Z., Liu F., Wu K., Zhong B. (2020). RNA based mNGS approach identifies a novel human coronavirus from two individual pneumonia cases in 2019 Wuhan outbreak. Emerg. Microbes Infect..

[21] Kilic T., Weissleder R., Lee H. (2020). Molecular and Immunological Diagnostic Tests of COVID-19: Current Status and Challenges. iScience.

[22] Deng H., Gao Z. (2015). Bioanalytical applications of isothermal nucleic acid amplification techniques. Anal. Chim. Acta.

[23] Suo T., Liu X., Feng J., Guo M., Hu W., Guo D., Ullah H., Yang Y., Zhang Q., Wang X. (2020). ddPCR: A more accurate tool for SARS-CoV-2 detection in low viral load specimens. Emerg. Microbes Infect..

[24] Jayamohan H., Lambert C.J., Sant H.J., Jafek A., Patel D., Feng H., Beeman M., Mahmood T., Nze U., Gale B.K. (2021). SARS-CoV-2 pandemic: A review of molecular diagnostic tools including sample collection and commercial response with associated advantages and limitations. Anal. Bioanal. Chem..

[25] Fomsgaard A.S., Rosenstierne M.W. (2020). An alternative workflow for molecular detection of SARS-CoV-2—Escape from the NA extraction kit-shortage, Copenhagen, Denmark, March 2020. Eurosurveillance.

[26] Williams E., Bond K., Zhang B., Putland M., Williamson D.A. (2020). Saliva as a noninvasive specimen for detection of SARS-CoV-2. J. Clin. Microbiol..

[27] La Rosa G., Iaconelli M., Mancini P., Bonanno Ferraro G., Veneri C., Bonadonna L., Lucentini L., Suffredini E. (2020). First detection of SARS-CoV-2 in untreated wastewaters in Italy. Sci. Total Environ..

[28] Bivins A., Greaves J., Fischer R., Yinda K.C., Ahmed W., Kitajima M., Munster V.J., Bibby K. (2020). Persistence of SARS-CoV-2 in Water and Wastewater. Environ. Sci. Technol. Lett..

[29] Razzini K., Castrica M., Menchetti L., Maggi L., Negroni L., Orfeo N.V., Pizzoccheri A., Stocco M., Muttini S., Balzaretti C.M. (2020). SARS-CoV-2 RNA detection in the air and on surfaces in the COVID-19 ward of a hospital in Milan, Italy. Sci. Total Environ..

[30] Zanoli L.M., Spoto G. (2013). Isothermal amplification methods for the detection of nucleic acids in microfluidic devices. Biosensors.

[31] Asadi R., Mollasalehi H. (2021). The mechanism and improvements to the isothermal amplification of nucleic acids, at a glance. Anal. Biochem..

[32] Panno S., Matić S., Tiberini A., Caruso A.G., Bella P., Torta L., Stassi R., Davino S. (2020). Loop mediated isothermal amplification: Principles and applications in plant virology. Plants.

[33] Li J.J., Xiong C., Liu Y., Liang J.S., Zhou X.W. (2016). Loop-mediated isothermal amplification (LAMP): Emergence as an alternative technology for herbal medicine identification. Front. Plant Sci..

[34] Pang B., Yao S., Xu K., Wang J., Song X., Mu Y., Zhao C., Li J. (2019). A novel visual-mixed-dye for LAMP and its application in the detection of foodborne pathogens. Anal. Biochem..

[35] Wang Y., Feng J., Tian X. (2019). Application of loop-mediated isothermal amplification (LAMP) for rapid detection of Atlantic cod (*Gadus morhua*), Pacific cod (*Gadus macrocephalus*) and haddock (*Melanogrammus aeglefinus*). Mol. Cell. Probes.

[36] Hongwarittorrn I., Chaichanawongsaroj N., Laiwattanapaisal W. (2017). Semi-quantitative visual detection of loop mediated isothermal amplification (LAMP)-generated DNA by distance-based measurement on a paper device. Talanta.

[37] Meena P.N., Kharbikar L.L., Rana R.S., Satpathy S., Shanware A., Sivalingam P.N., Nandanwar S. (2019). Detection of Mesta yellow vein mosaic virus (MeYVMV) in field samples by a loop-mediated isothermal amplification reaction. J. Virol. Methods.

[38] Zhou Y., Wan Z., Yang S., Li Y., Li M., Wang B., Hu Y., Xia X., Jin X., Yu N. (2019). A mismatch-tolerant reverse transcription loop-mediated isothermal amplification method and its application on simultaneous detection of all four serotype of dengue viruses. Front. Microbiol..

[39] Lu R., Wu X., Wan Z., Li Y., Zuo L., Qin J., Jin X., Zhang C. (2020). Development of a Novel Reverse Transcription Loop-Mediated Isothermal Amplification Method for Rapid Detection of SARS-CoV-2. Virol. Sin..

[40] Haque M.F.U., Bukhari S.S., Ejaz R., Zaman F.U., Sreejith K.R., Rashid N., Umer M., Shahzad N. (2021). A novel RdRp-based colorimetric RT-LAMP assay for rapid and sensitive detection of SARS-CoV-2 in clinical and sewage samples from Pakistan. Virus Res..

[41] Mohon A.N., Oberding L., Hundt J., van Marle G., Pabbaraju K., Berenger B.M., Lisboa L., Griener T., Czub M., Doolan C. (2020). Optimization and clinical validation of dual-target RT-LAMP for SARS-CoV-2. J. Virol. Methods.

[42] Lau H.Y., Botella J.R. (2017). Advanced DNA-based point-of-care diagnostic methods for plant diseases detection. Front. Plant Sci..

[43] Tian B., Gao F., Fock J., Dufva M., Hansen M.F. (2020). Homogeneous circle-to-circle amplification for real-time optomagnetic detection of SARS-CoV-2 RdRp coding sequence. Biosens. Bioelectron..

[44] Jiao J., Duan C., Xue L., Liu Y., Sun W., Xiang Y. (2020). DNA nanoscaffold-based SARS-CoV-2 detection for COVID-19 diagnosis. Biosens. Bioelectron..

[45] Haes A.J., Giordano B.C., Collins G.E. (2006). Aptamer-based detection and quantitative analysis of ricin using affinity probe capillary electrophoresis. Anal. Chem..

[46] Proske D., Blank M., Buhmann R., Resch A. (2005). Aptamers—Basic research, drug development, and clinical applications. Appl. Microbiol. Biotechnol..

[47] Platella C., Riccardi C., Montesarchio D., Roviello G.N., Musumeci D. (2017). G-quadruplex-based aptamers against protein targets in therapy and diagnostics. Biochim. Biophys. Acta Gen. Subj..

[48] Woo C.H., Jang S., Shin G., Jung G.Y., Lee J.W. (2020). Sensitive fluorescence detection of SARS-CoV-2 RNA in clinical samples via one-pot isothermal ligation and transcription. Nat. Biomed. Eng..

[49] Gootenberg J.S., Abudayyeh O.O., Lee J.W., Essletzbichler P., Dy A.J., Joung J., Verdine V., Donghia N., Daringer N.M., Freije C.A. (2017). Nucleic acid detection with CRISPR-Cas13a/C2c2. Science.

[50] Ding X., Yin K., Li Z., Lalla R.V., Ballesteros E., Sfeir M.M., Liu C. (2020). Ultrasensitive and visual detection of SARS-CoV-2 using all-in-one dual CRISPR-Cas12a assay. Nat. Commun..

[51] Myhrvold C., Freije C.A., Gootenberg J.S., Abudayyeh O.O., Metsky H.C., Durbin A.F., Kellner M.J., Tan A.L., Paul L.M., Parham L.A. (2018). Field-deployable viral diagnostics using CRISPR-Cas13. Science.

[52] Broughton J.P., Deng X., Yu G., Fasching C.L., Servellita V., Singh J., Miao X., Streithorst J.A., Granados A., Sotomayor-Gonzalez A. (2020). CRISPR–Cas12-based detection of SARS-CoV-2. Nat. Biotechnol..

[53] Patchsung M., Jantarug K., Pattama A., Aphicho K., Suraritdechachai S., Meesawat P., Sappakhaw K., Leelahakorn N., Ruenkam T., Wongsatit T. (2020). Clinical validation of a Cas13-based assay for the detection of SARS-CoV-2 RNA. Nat. Biomed. Eng..

[54] Microbiol C., Rabaan A.A., Ahmed S.H.A., Sah R., Tiwari R., Yatoo M.I., Patel S.K., Pathak M., Malik Y.S., Dhama K. (2020). SARS-CoV-2/COVID-19 and advances in developing potential therapeutics and vaccines to counter this emerging pandemic. Ann. Clin. Microbiol. Antimicrob..

[55] Wu D., Wu T., Liu Q., Yang Z. (2020). The SARS-CoV-2 outbreak: What we know. Int. J. Infect. Dis..

[56] Michael-Kordatou I., Karaolia P., Fatta-Kassinos D. (2020). Sewage analysis as a tool for the COVID-19 pandemic response and management: The urgent need for optimised protocols for SARS-CoV-2 detection and quantification. J. Environ. Chem. Eng..

[57] Ghodake G.S., Shinde S.K., Kadam A.A., Saratale R.G., Saratale G.D., Syed A., Elgorban A.M., Marraiki N., Kim D.Y. (2021). Biological characteristics and biomarkers of novel SARS-CoV-2 facilitated rapid development and implementation of diagnostic tools and surveillance measures. Biosens. Bioelectron..

[58] Ben-Ami R., Klochendler A., Seidel M., Sido T., Gurel-Gurevich O., Yassour M., Meshorer E., Benedek G., Fogel I., Oiknine-Djian E. (2020). Large-scale implementation of pooled RNA extraction and RT-PCR for SARS-CoV-2 detection. Clin. Microbiol. Infect..

[59] Ren K., Zhou J., Wu H. (2013). Materials for microfluidic chip fabrication. Acc. Chem. Res..

[60] Jiang X., Shao N., Jing W., Tao S., Liu S., Sui G. (2014). Microfluidic chip integrating high throughput continuous-flow PCR and DNA hybridization for bacteria analysis. Talanta.

[61] He S., Joseph N., Feng S., Jellicoe M., Raston C.L. (2020). Application of microfluidic technology in food processing. Food Funct..

[62] Park S., Zhang Y., Lin S., Wang T.H., Yang S. (2011). Advances in microfluidic PCR for point-of-care infectious disease diagnostics. Biotechnol. Adv..

[63] Ji M., Xia Y., Loo J.F.C., Li L., Ho H.P., He J., Gu D. (2020). Automated multiplex nucleic acid tests for rapid detection of SARS-CoV-2, influenza A and B infection with direct reverse-transcription quantitative PCR (dirRT-qPCR) assay in a centrifugal microfluidic platform. RSC Adv..

[64] Trinh T.N.D., La H.C., Lee N.Y. (2019). Fully Integrated and Foldable Microdevice Encapsulated with Agarose for Long-Term Storage Potential for Point-of-Care Testing of Multiplex Foodborne Pathogens. ACS Sens..

[65] Mitsakakis K., D’Acremont V., Hin S., von Stetten F., Zengerle R. (2018). Diagnostic tools for tackling febrile illness and enhancing patient management. Microelectron. Eng..

[66] Ducrée J., Haeberle S., Lutz S., Pausch S., Von Stetten F., Zengerle R. (2007). The centrifugal microfluidic Bio-Disk platform. J. Micromechanics Microengineering.

[67] Kang B.H., Lee Y., Yu E.S., Na H., Kang M., Huh H.J., Jeong K.H. (2021). Ultrafast and Real-Time Nanoplasmonic On-Chip Polymerase Chain Reaction for Rapid and Quantitative Molecular Diagnostics. ACS Nano.

[68] Soares R.R.G., Akhtar A.S., Pinto I.F., Lapins N., Barrett D., Sandh G., Yin X., Pelechano V., Russom A. (2021). Sample-to-answer COVID-19 nucleic acid testing using a low-cost centrifugal microfluidic platform with bead-based signal enhancement and smartphone read-out. Lab Chip.

[69] Rodriguez-Mateos P., Ngamsom B., Walter C., Dyer C.E., Gitaka J., Iles A., Pamme N. (2021). A lab-on-a-chip platform for integrated extraction and detection of SARS-CoV-2 RNA in resource-limited settings. Anal. Chim. Acta.

[70] Garneret P., Coz E., Martin E., Manuguerra J.C., Brient-Litzler E., Enouf V., González Obando D.F., Olivo-Marin J.C., Monti F., van der Werf S. (2021). Performing point-of-care molecular testing for SARS-CoV-2 with RNA extraction and isothermal amplification. PLoS ONE.

[71] Rodriguez-Manzano J., Malpartida-Cardenas K., Moser N., Pennisi I., Cavuto M., Miglietta L., Moniri A., Penn R., Satta G., Randell P. (2021). Handheld point-of-care system for rapid detection of SARS-CoV-2 extracted RNA in under 20 min. ACS Cent. Sci..

[72] Kim H.S., Abbas N., Shin S. (2021). A rapid diagnosis of SARS-CoV-2 using DNA hydrogel formation on microfluidic pores. Biosens. Bioelectron..

[73] Park J.S., Hsieh K., Chen L., Kaushik A., Trick A.Y., Wang T.H. (2021). Digital CRISPR/Cas-Assisted Assay for Rapid and Sensitive Detection of SARS-CoV-2. Adv. Sci..

[74] Burmeister A., Grünberger A. (2020). Microfluidic cultivation and analysis tools for interaction studies of microbial co-cultures. Curr. Opin. Biotechnol..

[75] Wang X., Liu Z., Pang Y. (2017). Concentration gradient generation methods based on microfluidic systems. RSC Adv..

[76] Wu J., Kodzius R., Cao W., Wen W. (2014). Extraction, amplification and detection of DNA in microfluidic chip-based assays. Microchim. Acta.

[77] Hu F., Li J., Zhang Z., Li M., Zhao S., Li Z., Peng N. (2020). Smartphone-Based Droplet Digital LAMP Device with Rapid Nucleic Acid Isolation for Highly Sensitive Point-of-Care Detection. Anal. Chem..

[78] Ye X., Li Y., Fang X., Kong J. (2020). Integrated Microfluidic Sample-to-Answer System for Direct Nucleic Acid-Based Detection of Group B Streptococci in Clinical Vaginal/Anal Swab Samples. ACS Sens..

[79] Zhong J., Zhao X. (2018). Isothermal Amplification Technologies for the Detection of Foodborne Pathogens. Food Anal. Methods.

[80] Barreda-García S., Miranda-Castro R., de-los-Santos-Álvarez N., Miranda-Ordieres A.J., Lobo-Castañón M.J. (2018). Helicase-dependent isothermal amplification: A novel tool in the development of molecular-based analytical systems for rapid pathogen detection. Anal. Bioanal. Chem..

[81] Zhao Y., Chen F., Li Q., Wang L., Fan C. (2015). Isothermal Amplification of Nucleic Acids. Chem. Rev..

[82] Suea-Ngam A., Bezinge L., Mateescu B., Howes P.D., Demello A.J., Richards D.A. (2020). Enzyme-Assisted Nucleic Acid Detection for Infectious Disease Diagnostics: Moving toward the Point-of-Care. ACS Sens..

[83] Li M., Ge A., Liu M., Ma B., Ma C., Shi C. (2020). A fully integrated hand-powered centrifugal microfluidic platform for ultra-simple and non-instrumental nucleic acid detection. Talanta.

[84] Yu W., Chen Y., Wang Z., Qiao L., Xie R., Zhang J., Bian S., Li H., Zhang Y., Chen A. (2021). Multiple authentications of high-value milk by centrifugal microfluidic chip-based real-time fluorescent LAMP. Food Chem..

[85] Yuan D., Kong J., Li X., Fang X., Chen Q. (2018). Colorimetric LAMP microfluidic chip for detecting three allergens: Peanut, sesame and soybean. Sci. Rep..

[86] Zhou Q.J., Wang L., Chen J., Wang R.N., Shi Y.H., Li C.H., Zhang D.M., Yan X.J., Zhang Y.J. (2014). Development and evaluation of a real-time fluorogenic loop-mediated isothermal amplification assay integrated on a microfluidic disc chip (on-chip LAMP) for rapid and simultaneous detection of ten pathogenic bacteria in aquatic animals. J. Microbiol. Methods.

[87] Dhar B.C., Lee N.Y. (2018). Lab-on-a-Chip Technology for Environmental Monitoring of Microorganisms. Biochip J..

[88] Zhou X., Liu D., Zhong R., Dai Z., Wu D., Wang H., Du Y., Xia Z., Zhang L., Mei X. (2004). Determination of SARS-coronavirus by a microfluidic chip system. Electrophoresis.

